# Dental Trauma Prevention and Injury Measures Among Supervisors of German Elite Handball Teams: A Questionnaire‐Based Cross‐Sectional Study

**DOI:** 10.1002/cre2.70217

**Published:** 2025-09-08

**Authors:** Kyra Beatrice Kurz, Theresa Antonia Rott, Dirk Ziebolz, René Toussaint

**Affiliations:** ^1^ Department of Cariology, Endodontology and Periodontology University of Leipzig Leipzig Germany; ^2^ Department of Conservative Dentistry and Periodontology Brandenburg Medical School Theodor Fontane (MHB) Brandenburg a.d. Havel Germany; ^3^ Institute for General Practice, Faculty of Medicine Leipzig University; ^4^ Medical Centre for Orthopaedics and Sports Dentistry Leipzig Germany

**Keywords:** athletes, basketball, dental trauma, handball, prevention, sports dentistry, sports mouthguards

## Abstract

**Objectives:**

Dental trauma is a frequent injury in contact sports such as handball an basketball. This study aimed to evaluate preventive measures in dental traumatology and assess the knowledge of medical teams in elite German handball and basketball.

**Material and Methods:**

From March to June 2024, supervisors of 1st and 2nd German Bundesliga handball (HB) and basketball (BB) teams were invited via email to complete an online questionnaire (Socey Survey). The survey addressed preventive measures, management of dental and dentoalveolar trauma, the importance of education, and behavioral guidelines.

**Results:**

A total of 31 of 165 supervisors participated (response rate 19%; HB: 42% [*n* = 28], BB: 3% [*n* = 3]). Analyses focused on HB due to the limited number of BB responses. Most HB supervisors (82%) reported checking tetanus immunization status. Although 39% recommended sports mouthguards, only 15% of players used them. During the 2022/2023 season, 29% of HB teams reported at least one dental trauma. Although 96% recognized the need for immediate dental care in pulp‐involved trauma, only 64% were aware of dental rescue boxes, and 46% lacked one on the pitch. Clear differences were observed between leagues: 82% of 1st Bundesliga teams had a rescue box compared to 11% in the 2nd Bundesliga. Mouthguard use was also more common in the 1st Bundesliga.

**Conclusions:**

In elite German handball, dental trauma prevention focuses primarily on tetanus vaccination, whereas sports mouthguards and dental rescue boxes receive less attention. Targeted education and awareness campaigns for coaches and players may improve the prevention, management, and treatment of dental injuries.

## 1 Introduction

A considerable number of oral and dental injuries occur in contact sports, including American football, basketball, rugby, football, handball, and boxing (Bourguignon and Sigurdsson 2009; Zürcher et al. 2020; Petrovic et al. 2016), all of which are associated with a higher risk of traumatic dental injuries (Dursun et al. 2015). In handball (HB) and basketball (BB), these injuries are typically caused by collisions with other players, resulting in blows to the facial area with hands or elbows, or by a direct hit from the ball at close range (Bergman et al. 2017).

In severe cases, dental or dentoalveolar trauma may result in the immediate loss of the affected tooth (e.V. BZÄK B‐A der DZ 2024; Brüllmann and d'Hoedt 2017). Even if the tooth is preserved, permanent damage such as pulp necrosis, tooth fractures, external root resorption, or ankylosis may still occur. These conditions often require complex and costly treatment (Wang et al. 2014; Andreasen et al. 2012; Andersson 2013), and can also ultimately lead to tooth loss (Finucane and Kinirons 2003; Patel et al. 2022). A dental trauma can also impact an athlete's oral health‐related quality of life, as it may result in both pain and functional limitations, such as difficulties with eating and speaking (Das et al. 2022; Merle et al. 2024).

For this reason, prevention and the implementation of suitable measures to avoid dental trauma are of great importance (de Gregorio and Tewari 2024). Wearing a sports mouthguard during sporting activities is considered an effective measure for preventing dental trauma (Newsome et al. 2001; Knapik et al. 2019). The mouthguard reduces the direct force of an impact and protects teeth, jawbones, and surrounding soft tissue structures from injury (Dhillon et al. 2014; Knapik et al. 2007). A current systematic review revealed the prevalence of dental injuries in contact sports of around 8% among athletes who utilized mouthguards, and between 48% and 60% in athletes without mouthguards (Fernandes et al. 2019). It can be accepted that in full‐contact sports, such as rugby, wearing a mouthguard is mandatory during competition (Deutscher Rugby Verband 2014); a reduction of dental injuries can be determined (Dhillon et al. 2014). HB and BB are classified as medium‐risk sports for dental injuries according to the *Fédération Dentaire International* (FDI) (Merglova 2018). However, the use of sports mouthguards in these disciplines is low (2% to 4%) compared to other contact sports (hockey: 91%, jiu‐jitsu: 20%, and judo: 7%) (Ferrari and De Medeiros 2002).

It is also important to educate athletes, coaches, and medical supervisors, as well as parents, about the risks of dental injuries and the significance of mouthguards (Petrovic et al. 2016; Ozbay et al. 2013; Hacquin et al. 2021; Ma 2008; Perunski et al. 2005a; Emerich and Kaczmarek 2010; Keçeci et al. 2005; Lang et al. 2002; Kumamoto and Maeda 2004). In addition to preventive measures, proper initial treatment is crucial to minimize the harm caused by dental trauma (Nolte et al. 2023).

The tooth rescue box, a storage container filled with a cell culture medium designed to preserve the periodontal ligament cells of avulsed teeth, provides optimal storage until replantation (Pohl et al. 2005) and should therefore be available in sports (Filippi et al. 2008).

Whether these issues are addressed among athletes in Germany remains unanswered. Moreover, the implementation of preventive and treatment measures for tooth‐related injuries in elite HB and BB in Germany remains unclear, as does the extent of general awareness regarding available preventive options for dentoalveolar trauma.

The aim of this questionnaire‐based cross‐sectional study was to determine the current level of knowledge among medical care supervisors of German elite HB and BB teams regarding preventive and primary dental (trauma) care measures.

It was hypothesized that the use of sports mouthguards is rarely recommended in German elite HB and BB. Additionally, it was postulated that, although the use of tooth rescue boxes for initial treatment is widely known, they are seldom utilized on the pitch in elite HB and BB leagues in cases of dental trauma.

## 2 Methods

### 2.1 Study Design

This questionnaire‐based cross‐sectional study covers various aspects concerning the prevention of and intervention in cases of dental/dentoalveolar trauma in German elite handball (HB) and basketball (BB). The study was approved by the local Ethics Committee of the medical faculty of Leipzig University, Germany (No. 118/24‐ek). All participants who completed the questionnaire anonymously consented to participate in the study.

### 2.2 Data Collection and Questionnaire

The questionnaire covered topics related to the prevention, treatment, and detection of dental and dentoalveolar trauma, as well as the importance of trauma prevention education and behavioral guidelines. It also addressed the implementation of preventive measures (e.g., tetanus vaccination status, sports mouthguards, dental rescue boxes, and education).

The questionnaire comprised 26 questions with a variety of question formats, including multiple‐choice, drop‐down selections, and closed‐ and open‐ended questions. The questions were grouped into three categories: socio‐demographic, action‐related, and trauma‐related questions. Basic information such as age and gender was collected in the socio‐demographic section. The action‐related questions assessed theoretical knowledge through case scenarios, covering topics such as tooth storage and appropriate procedures after dental trauma. The trauma‐related questions focused on the practical implementation of prevention and first aid measures, addressing aspects such as injury frequency and mouthguard use (Table S1).

The questionnaire was created using the SoSci Survey web application (www.soscisurvey.de; SoSci Survey GmbH, Munich, Germany).

In December 2023, a pretest with five physicians was conducted to assess the feasibility of the questionnaire.

### 2.3 Participants

The respondents to the survey were medical care supervisors working in healthcare for the 1st and 2nd German HB and BB *Bundesliga* (men and women). These supervisors, who include professionals such as doctors, physiotherapists, and dentists, were required to have provided medical support for at least 1 month in the 1st or 2nd German *Bundesliga* in either HB or BB. The inclusion criteria also specified that respondents must be at least 18 years old. In accordance with the objective criteria established for this selection process, supervisors from clubs in the third national HB or BB league, or lower leagues, were not included in the survey.

Between March and June 2024, the medical care supervisors of HB and BB teams in the 1st and 2nd *Bundesliga* were invited by email to participate in an online questionnaire. The email addresses of the medical supervisors, which can be found on the websites of the 1st and 2nd *Bundesliga* in HB and BB, were used to send the online questionnaire via an invitation link. After 6 weeks, the participants received a reminder/reactivation email with the same invitation link. The online survey was conducted anonymously, without collecting personal data such as names or email addresses of the participants.

### 2.4 Background Player Analysis

To analyze the cohort that the questionnaire focuses on (players from the 1st and 2nd *Bundesliga* in HB and BB), we retrieved data on team size, players' nationalities, and ages from the official club websites.

### 2.5 Data Preparation

All source materials originally in German were translated into English before analysis. The translation process was supported by the DeepL online tool, and all outputs were subjected to thorough manual review and revision to ensure linguistic and conceptual accuracy.

### 2.6 Statistical Analysis

Descriptive statistics were calculated for categorical and metric variables. Frequencies are presented for categorical data, whereas means and standard deviations are presented for continuous variables. Pearson correlation coefficients (*r*) were used to examine associations between relevant variables. Correlation strengths were interpreted using conventional thresholds: weak (*r* < 0.3), moderate (*r* = 0.3–0.6), and strong (*r* > 0.6). Statistical analysis was performed using SPSS for Windows, version 29.0 (IBM, USA).

## 3 Results

### 3.1 Response Rate

A total of twenty eight (42%) of the 66 HB teams and three (3%) of the 99 BB teams in the 1st and 2nd *Bundesliga* responded with a fully completed questionnaire, resulting in an overall response rate of 19%.

### 3.2 Study Participants

In the current cross‐sectional study, a total of 31 questionnaires were included. A detailed overview of the characteristics of the participants from the areas of HB and BB is shown in Table 1. Given the limited number of BB supervisors who participated in the study, the subsequent analysis of the questionnaires will focus on the responses from the HB supervisors. In this field, two‐thirds of the participants who answered the questionnaire were physicians. More than half of them (54%) were specialists in orthopedics and trauma surgery, 12% were internists, and 4% were in general practice. The data revealed that half of the HB clubs (50%) had a dental contact (i.e., a designated dentist responsible for the team, available for consultation, and possibly present at events).

**Table 1:** Characteristics of the respondents.

		Basketball	Handball
Number of respondents [*n* (%)]		3 (100)	28 (100)
Gender female [*n* (%)]		1 (33)	6 (21)
Age in years [*n* (%)]	18–24	0 (0)	0 (0)
	25–34	1 (33)	4 (14)
	35–50	2 (67)	8 (29)
	> 50	0 (0)	16 (57)
Club League [*n* (%)]	1st MB	2 (67)	11 (39)
	1st WB	0 (0)	8 (29)
	2nd MB	1 (33)	4 (14)
	2nd WB	0 (0)	5 (18)
Duration of support in years [*n* (%)]	≤ 5	2 (67)	9 (32)
	> 5	1 (33)	19 (68)

Abbreviations: *n*, number of respondents; 1st MB, Men's 1st Bundesliga; 1st WB, Women's 1st Bundesliga; 2nd MB, Men's 2nd Bundesliga; 2nd WB, Women's 2nd Bundesliga.

### 3.3 Analysis of the Players

**Figure 1:**
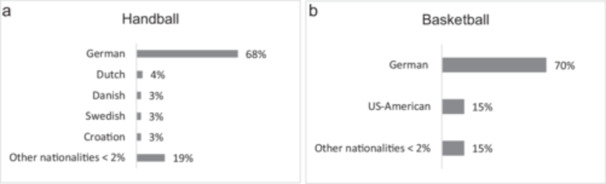
(a, b) Most common nationalities in elite handball and basketball. Nationalities that represent less than two percent of the total were summarized under “other nationalities.”

In order to establish the potential cohort to be supervised, all teams in the 1st and 2nd *Bundesliga* in HB and BB for the 2022/2023 season were subjected to analysis. The cohort comprised 2689 players (HB: *n* = 1222, BB: *n* = 1467), with an average age of 25.1 years in HB and 23.9 years in BB. Both sports show a certain heterogeneity regarding players' nationalities. Approximately two‐thirds of the athletes in both disciplines are of German descent. In HB, the majority of players are from European countries (97%), whereas in BB, approximately 15% of players are from the USA (Figure 1a,b).

### 3.4 Action‐Related Questions

**Figure 2:**
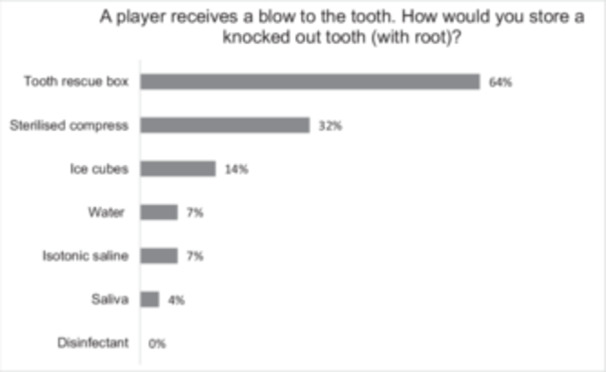
Supervisors of the 1st and 2nd German handball Bundesliga were asked about tooth storage media. The participants were able to choose more than one possible answer.

The detailed responses to the action‐related questions are presented in Table 2 and Figure 2. *Case 1:* Approximately 71% of supervisors reported checking their players' tetanus vaccination status for dentoalveolar trauma prevention. Among medical care supervisors, doctors (84% of 19 doctors) most commonly performed this check. *Case 2:* Regarding the storage of a knocked‐out tooth, the tooth rescue box was chosen as the most frequently indicated storage medium (64%). Other selected storage options are shown in Figure 2. *Case 3:* The majority (96%) recognized the need to consult a dentist immediately after dental trauma involving pulp exposure. *Case 4:* Over half (61%) of the cohort would seek dental care on the next working day if a player's tooth corner were fractured (Table 2).

**Table 2:** Supervisors of the 1st and 2nd German handball Bundesliga answered questions about dental trauma prevention and injury measures. The participants were able to choose more than one possible answer.

		HB supervisors (*n* = 28)
What do you ask about precautions regarding dentoalveolar trauma prevention? [*n* (%)]	Abscess anamnesis	7 (25)
	Tetanus immunization status	20 (71)
	Immunodeficiency diseases	13 (46)
	Antibiotic tolerance	16 (57)
	Implants	1 (4)
	Dental operations	1 (4)
	Dental injuries	1 (4)
	Dental status	1 (4)
If a player receives a blow to the tooth, resulting in a broken crown that is bleeding and the root is still in the socket, what would you do? [*n* (%)]	Clean the wound area with disinfectant	4 (14)
	Visit the dentist immediately	27 (96)
	Visit the dentist on the next working day	1 (4)
	Visit the dentist in case of pain	1 (4)
If a player receives a blow to the tooth, resulting in a broken corner of the tooth without bleeding, what would you do? [*n* (%)]	Clean the wound area with disinfectant	3 (11)
	Visit the dentist immediately	10 (36)
	Visit the dentist on the next working day	17 (61)
	Visit the dentist in case of pain	3 (11)

Abbreviations: HB, handball; *n*, number of respondents.

### 3.5 Trauma‐Related Questions

#### 3.5.1 Prevention Measures

##### 3.5.1.1 Prevention of Tetanus Infection

**Figure 3:**
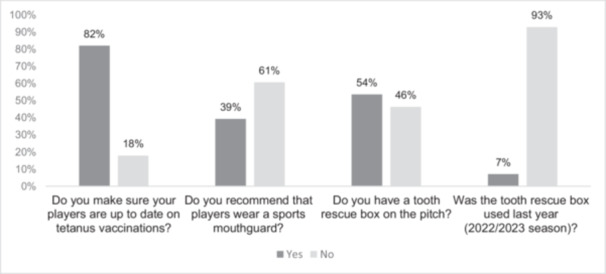
Supervisors of the 1st and 2nd German handball Bundesliga answered questions about dental trauma prevention and injury measures.

**Figure 4:**
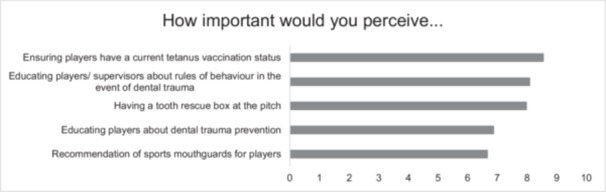
Questions of importance. Supervisors of the 1st and 2nd German handball Bundesliga rated the importance of various aspects of dental trauma prevention and injury measures. Ratings were made between 0 = unimportant and 10 = highly important.

The majority (82%) of respondents indicated that they pay attention to the tetanus immunization status of players (Figure 3). Checking the tetanus vaccination status is of the utmost importance for medical care supervisors (Figure 4 and Table 3).

**Table 3:** Addition of the standard deviation to Figure 4: Questions of importance. Supervisors of the 1st and 2nd German handball Bundesliga rated the importance of various aspects of dental trauma prevention and injury measures. Ratings were made between 0 = unimportant and 10 = highly important.

How important would you perceive …	±SD (*n* = 28)
Ensuring players have a current tetanus vaccination status	± 2.4
Educating players about dental trauma prevention	± 2.5
Having a tooth rescue box at the pitch	± 2.7
Educating players/supervisors about the rules of behavior in the event of dental trauma	± 2.9

Furthermore, 68% of the medical care supervisors (*n* = 19/28) were able to provide information on the number of athletes in their teams who had received up‐to‐date tetanus immunizations. According to their responses, 99% of these athletes (*n* = 351) had a current tetanus vaccination status.

##### 3.5.1.2 Prevention of Dental Trauma

Based on the information provided by medical care supervisors, 15% of all supervised players wore mouthguards (*n* = 79/511); on average, three players in the 1st *Bundesliga* and two in the 2nd *Bundesliga* used them. A moderate positive correlation was observed between league level and mouthguard usage (*r* = 0.5). The correlation analysis showed that more players in the higher league wore mouthguards.

Moreover, 39% (*n* = 11/28) of the participating HB supervisors recommended mouthguard use to their players (Figure 3). The results showed a strong positive correlation between the supervisors' recommendation of mouthguards and the number of players actually wearing them (*r* = 0.8). Teams whose supervisors more frequently recommended mouthguards had a higher number of players wearing them.

HB supervisors rated the importance of educating players on dental trauma prevention and recommending sports mouthguards between 6 and 7 points on a scale from 0 to 10 (Figure 4 and Table 2).

#### 3.5.2 Initial Treatment After Dental Trauma

A total of 29% of the participating HB clubs reported at least one case of dental trauma during the previous season (2022/2023).

Approximately half (54%) of the HB teams have a tooth rescue box near the pitch. It was used by 7% of the teams last season (Figure 3). A total of 36% stated that they did not know how or for what purpose a dental rescue box is used. The majority of teams in the men's 1st *Bundesliga* (82%) have a tooth rescue box, while teams in the 2nd *Bundesliga* (men and women) tend to have such boxes less frequently (89% do not have a tooth rescue box). Fewer tooth rescue boxes were available in the lower leagues (*r* = 0.6).

## 4 Discussion

### 4.1 Summary of the Main Results

HB (medical care) supervisors are more inclined to ascertain that players have a current tetanus immunization (82%), yet comparatively less likely to recommend the use of a sports mouthguard (39%). Less than two‐thirds (64%) know how and why to use a dental rescue box. Almost half (54%) of HB clubs have a tooth rescue box on the pitch, and 7% of teams used it during the previous season.

### 4.2 Interpretation in Comparison With the Available Literature

*Response rate and analysis of the players:* While players from European nations are predominant in the 1st and 2nd HB *Bundesliga*, the influence of US players is strong in BB, which is not surprising given the popularity of the sport in the United States (Sorbara 2022). The unexpectedly low response rate of BB supervisors (*n* = 3) allows for some speculation. It is conceivable that the influence of US players, who are already well informed about dental trauma through campaigns such as “Save a Tooth Poster” (Save a Tooth Poster 2024) or “Facial Protection Month” (National Facial Protection Month 2024) in the United States, means that coaches and/or medical care supervisors are less reliant on additional education. Similarly, the focus in BB may be on more common injuries, such as fractures, torn ligaments, and joint problems (Lian et al. 2022), which could lead to less attention being paid to dental injuries. Moreover, time and organizational constraints, particularly in higher leagues, may also have contributed to the low participation rate. The higher response rate observed in HB (42%) may be indicative of a heightened perceived necessity for educational initiatives concerning the prevention of dental trauma. One possible improvement would be to integrate the survey into existing education programs for coaches/medical care supervisors to facilitate participation and increase its relevance.

*Action‐related questions:* In the present study, the majority of supervisors (96%) recognized the urgency of immediate treatment for dental trauma involving the dental pulp. Similar results were found in a recent French study, in which 92% of HB players categorized a knocked‐out tooth as an emergency (Hacquin et al. 2021). In addition, the current study showed that 61% of supervisors correctly classified a chipped tooth corner as not a medical emergency. These results suggest that HB supervisors of the 1st and 2nd German HB *Bundesliga* are able to distinguish between serious dental injuries requiring immediate emergency treatment and less acute damage. In addition, this demonstrates that they have a basic understanding of the urgency of treatment.

*Prevention of tetanus infection:* Among the medical care supervisors who were able to provide information about their players' current tetanus immunization status, it was reported that 99% of players (*n* = 351 supervised players) were immunized. These results are based on the supervisors' assessments of their players' vaccination status, not on reports from the players themselves. Only clubs for which supervisors were able to provide information were included in the analysis. Notably, 32% of the supervisors were unable to provide any information about their players' vaccination status. The prevalence of the current tetanus immunization status of HB players determined in the current study is therefore notably higher than the tetanus immunization rate of 73% determined for the adult population in Germany in a study from 2011 (Böhmer et al. 2011). A comparison with available studies also demonstrates the high prevalence of the data presented here. According to an Italian study, 73% (*n* = 270) of amateur BB players had received the vaccination (Riccò and Peruzzi 2022), whereas in the United States, only one in six high school senior athletes had received the recommended tetanus vaccination (Karpinos et al. 2014). However, the number of studies investigating the tetanus immunization status of athletes is limited, highlighting the need for further research.

The result of the present study has shown a tendency for medical care supervisors in particular to pay attention to tetanus immunization status, presumably due to their extensive training in infectious diseases and their prevention. Team doctors/medical care supervisors play a key role, as they not only provide medical care to athletes, but can also make sound immunization recommendations (Boston and Bryan 2018). Supervisors in other areas should also be informed about the increased risk of tetanus infection to pay particular attention to this. It is notable that respondents indicated that their tetanus immunization status was of particular importance to them, in comparison to other forms of prevention and primary care measures (Figure 4, Table 2). This is also reflected in the fact that the majority (82%) of respondents indicated that they consider the immunization status of players (Figure 3).

*Prevention of dental trauma:* The current study demonstrated that approximately 15% utilize a mouthguard. The data on the prevalence of sports mouthguard use in the literature varies considerably, depending on the level of performance and country of origin, ranging from 0% to 28% (Zürcher et al. 2020; Petrovic et al. 2016; Bergman et al. 2017; Ferrari and De Medeiros 2002; Ozbay et al. 2013; Keçeci et al. 2005; Hacquin et al. 2021; Perunski et al. 2005b; Hwang and Kim 2019). A comparable Swiss study, in which players and supervisors in the elite HB leagues were surveyed on the prevention of dental trauma, revealed that 6% (*n* = 29/507) of players wear a mouthguard (Petrovic et al. 2016). The prevalence of the use of mouthguards in elite German sports was investigated in a recent study, which surveyed athletes from 30 different sports using a questionnaire. Of those surveyed, only 4% (*n* = 13/337) reported owning a mouthguard, while 10% reported being informed about the importance of mouthguards (Merle et al. 2024). To date, there is no comparable data from studies conducted in Germany for the sports of HB and BB. In the Croatian study with the highest prevalence (28%) of wearing a mouthguard in elite HB, 77% of these players also stated that they had been advised to wear a mouthguard (Bergman et al. 2017). The discrepancy in the wearing of mouthguards in the different countries could be due to the implementation of different prevention strategies or educational measures. Although almost 30% of the participating HB teams in this study were affected by dental trauma in the previous season, less than half of the supervisors in Germany recommended wearing a mouthguard (Figure 3).

In the current study, the correlation between supervisor recommendations and mouthguard use shows that players are more likely to adopt protective measures when guided by medical staff—underlining the important role of supervisor communication in injury prevention.

*Initial treatment after dental trauma:* A total of 64% of respondents stated that they would utilize a tooth rescue box (which contains a nutrient medium with salts, amino acids, glucose, and vitamins; IS Khinda et al. 2017) for the storage of an avulsed tooth. In comparison, saline solution was used most frequently in France (39%) (Hacquin et al. 2021) and a handkerchief (25%) in Turkey (Sepet et al. 2014). In the absence of a tooth rescue box, alternatives such as milk isotonic saline or saliva can be used (Fouad et al. 2020). Nevertheless, the current study showed that sterile compresses (32%) were the second most prevalent medium, followed by ice cubes (14%) and water (7%), all of which are unsuitable for storage (Poi et al. 2013; IS Khinda et al. 2017). Almost two‐thirds (64%) of participating supervisors knew how to use a tooth rescue box. This is a significantly higher proportion compared to the results of studies in HB and BB in Switzerland and Germany, where players/supervisors were surveyed (6%–8%) (Petrovic et al. 2016; Emerich and Kaczmarek 2010; Lang et al. 2002; Perunski et al. 2005b). Studies in sports like rugby, squash, and floorball show similar results regarding the knowledge of the tooth rescue box (5%–7%) (Schildknecht et al. 2012; Persic et al. 2006; Maxén et al. 2011). While the use of tooth rescue boxes is widespread in Switzerland and Germany, it appears to be less common in countries such as France and Sweden (Persic et al. 2006; Maxén et al. 2011). Given the scarcity of comparable literature from other countries, it seems likely that the tooth rescue box is also less known internationally.

Despite respondents indicating the significance of a tooth rescue box and the necessity of disseminating information regarding protocols for dental trauma, with an average rating of nearly 8 on a scale of 0 to 10, only approximately half of the HB clubs (54%) currently have such a box available. There is no available literature regarding the presence of tooth rescue boxes in other sports clubs. These results indicate significant gaps in practical implementation, suggesting a lack of equipment and a clear need for training to respond appropriately in emergencies.

### 4.3 Differences Between the 1st and 2nd HB Bundesliga

The current study revealed differences in equipment between the 1st and 2nd HB *Bundesliga*: A total of 82% of participating teams in the 1st men's HB *Bundesliga* have a tooth rescue box, whereas only 11% of teams in the 2nd *Bundesliga* (men's and women's) do. Furthermore, the utilization of mouthguards was observed to be more prevalent in the 1st Bundesliga (*r* = 0.5). It seems reasonable to posit that the elevated risk of dental trauma observed in professional athletes relative to semi‐professionals and amateurs (Ma 2008; Gassner et al. 1999) also exists when comparing the 1st and 2nd HB *Bundesliga*. It can be assumed that the increased availability of dental rescue boxes and mouthguards may be due to the higher frequency of dental injuries and greater awareness among supervisors in the first league. Given the large number of international players in the 1st HB *Bundesliga*, it is possible that players from certain nations, such as Croatia (Bergman et al. 2017), may be more inclined to wear mouthguards. Conversely, in the lower leagues, financial limitations and a lack of awareness regarding the importance of such measures may be contributing factors.

### 4.4 Strengths and Limitations

A key strength of this study is its focus on elite athletes, a group often underrepresented in sports health research on oral health, providing valuable insights into the prevention and management of dental trauma in high‐performance sports. However, there are only a few comparable studies in the sports of HB and BB in Germany and other countries. Additionally, the structured, web‐based questionnaire facilitated a systematic collection of quantitative data on supervisors' knowledge, attitudes, and practices, ensuring methodological rigor and reliability in the findings. Furthermore, the response rate (42%) among HB supervisors allows for solid statistical analyses, enhancing the relevance of findings for this sport.

However, the study also faces some limitations: The low participation rate among BB supervisors restricts the generalizability of findings to this sport, which limits a comprehensive comparison between HB and BB. Relying on self‐reported data introduces the risk of response bias, as participants might overestimate their adherence to preventive measures or their knowledge of dental trauma management. Furthermore, information on the prevention and initial management of dentoalveolar trauma is limited to the perspectives of medical supervisors, leaving a gap in the understanding of athletes' behaviors and potential barriers. Therefore, follow‐up studies involving athletes are warranted to assess their awareness, willingness, and compliance with dental trauma prevention and management measures. Additionally, email‐based recruitment could introduce sampling bias if certain supervisors were less inclined to participate, potentially skewing responses toward those more aware or supportive of dental health practices. Although the questionnaire was pre‐tested with five physicians to assess clarity and feasibility, no formal reliability testing (e.g., internal consistency or test‐retest reliability) was conducted, which constitutes a methodological limitation. The study's focus on German elite teams confines findings to this context, limiting applicability to international settings where preventive measures and awareness may differ. Finally, as a cross‐sectional study, it does not track changes over time in supervisors' knowledge or preventive practices, thus limiting insights into potential shifts in attitudes and behaviors.

## 5 Conclusions

In the 1st and 2nd German HB *Bundesliga*, dental trauma prevention focused primarily on tetanus vaccination, while recommendations for mouthguards and the availability of dental rescue boxes received less attention. This suggests limited dissemination and implementation of measures for the prevention and treatment of dental injuries, with uncertainty about the proper use of dental rescue boxes. Targeted education programs for coaches and players could improve awareness and response consistency. These findings highlight the need for more targeted information on dental trauma prevention and first aid treatment. To address these gaps, enhanced implementation recommendations are required, such as the promotion of mouthguards through league‐wide policies and the inclusion of first‐aid training modules.

## Author Contributions

All authors were involved in the conception, planning, and interpretation of the study. Material preparation, data collection, statistical analysis, and evaluation were carried out by Dirk Ziebolz, Theresa Antonia Rott, and Kyra Beatrice Kurz. The first draft of the manuscript was written by Theresa Antonia Rott and Kyra Beatrice Kurz. All authors critically revised earlier versions of the manuscript, discussed the content, and read and approved the final version.

## Conflicts of Interest

The authors declare no conflicts of interest.

## Supporting information


**Supplementary Table 1:** Summary of survey questions and formats.

## Data Availability

The datasets generated and analyzed during the current study are available from the corresponding author upon reasonable request.
