# Supplementary material for: Dental Trauma Prevention and Injury Measures Among Supervisors of German Elite Handball Teams: A Questionnaire‐Based Cross‐Sectional Study
Source: Clin Exp Dent Res. 2025 Sep 8;11(5):e70217. doi: 10.1002/cre2.70217 (PMC12415705; doi:10.1002/cre2.70217)
Supplement: Supplementary file 1 — Supplementary Table 1: Summary of survey questions and formats. [file CRE2-11-e70217-s001.docx]

**Supplementary Material**

Supplementary Table 1. Summary of survey questions and formats

| **Part/Item** |  | **Question** | **Question format** |
| --- | --- | --- | --- |
| **Socio-demographic** | | Which gender do you have? | Multiple choice |
|  |  | Which age group do you belong to? | Multiple choice |
|  |  | Which league does your club play in? | Multiple choice |
|  |  | How many players does the team have? | Closed-ended question |
|  |  | In which federal state is the club located? | Drop-down selection |
|  |  | What function do you have in the club? | Multiple choice |
|  |  | If you are a doctor, which residency you complete? | Drop-down selection |
|  |  | How long have you been supervising teams at the professional level? | Closed-ended question |
|  |  | Do you have a dental contact in the club? | Multiple choice  (yes or no) |
| **Action-related questions** | | What do you ask about dentoalveolar trauma prevention? | Multiple choice and open-ended question |
|  |  | A player suffers a blow to a tooth. The entire tooth (with root) is knocked out of the socket. How do you store the tooth? | Multiple choice and open-ended question |
|  |  | A player suffers a blow to a tooth, causing the crown to break off. The tooth bleeds, but the crown is still in the bone socket. What would you do? | Multiple choice and open-ended question |
|  |  | A player suffers a blow to a tooth, breaking off a corner of the crown. The tooth does not bleed. What would you do? | Multiple choice and open-ended question |
| **Trauma-related questions** | | How many dental/dentoalveolar traumas were reported to you by players last year (2022/2023 season) or did you detect? | Drop-down selection |
|  |  | How important is it to you that players are educated about dental trauma prevention? | Multiple choice (scale 0 to 10) |
|  |  | How important is education on behavioral guidelines for players and/or staff in cases of dentoalveolar trauma to you? | Multiple choice (scale 0 to 10) |
|  |  | Do you ensure that your players have an up-to-date tetanus vaccination? | Multiple choice  (yes or no) |
|  |  | How important do you consider ensuring that your players have an up-to-date tetanus vaccination? | Multiple choice (scale 0 to 10) |
|  |  | How many players on the team have a current tetanus immunisation status? | Drop-down selection |
|  |  | Do you recommend your players to wear a sports mouthguard? | Multiple choice  (yes or no) |
|  |  | How important do you consider recommending the use of a sports mouthguard to your players? | Multiple choice (scale 0 to 10) |
|  |  | How many players on the team have a sports mouthguard? | Drop-down selection |
|  |  | Do you have a tooth rescue box near the pitch? | Multiple choice  (yes or no) |
|  |  | How important do you consider having a tooth rescue box near the pitch? | Multiple choice (scale 0 to 10) |
|  |  | Was the tooth rescue box used in the past year (2022/2023 season)? | Multiple choice  (yes or no) |
|  |  | Do you know how and for what purpose a tooth rescue box is used? | Multiple choice  (yes or no) |
